# Gas Phase Toluene Adsorption Using Date Palm-Tree Branches Based Activated Carbon

**DOI:** 10.3390/ijerph17249287

**Published:** 2020-12-11

**Authors:** Muhammad Vohra, Mohammad Al-Suwaiyan, Minaam Hussaini

**Affiliations:** Environmental Engineering Program, Civil and Environmental Engineering Department, King Fahd University of Petroleum & Minerals (KFUPM), Dhahran 31261, Saudi Arabia; msaleh@kfupm.edu.sa (M.A.-S.); g201705690@kfupm.edu.sa (M.H.)

**Keywords:** activated carbon, toluene gas, adsorption, date palm-tree branches

## Abstract

Activated carbon that has been widely used for several environmental applications is typically produced from carbon-based raw materials including agricultural by-products. To that end, extensive date palm-tree farming across the globe with millions of palm trees, also results in various types of agricultural waste including date palm-tree branches (DPB) during the regular trimming phase of palm-trees. Furthermore, air pollution also remains a serious concern in many global regions, requiring the application of appropriate treatment technologies to mitigate the respective negative effects on human health and environment. The present study thus assessed the efficiency of activated carbon (AC) derived from date palm-tree branches to treat gaseous toluene (C_6_H_5_CH_3_) streams under varying dynamic flow conditions. The produced activated carbon showed BET specific surface area (*SSA*_BET_) of 800.87 m^2^/g with micro and mesoporous structure. The AC FTIR results indicated several surface groups including oxygen based functional groups. Furthermore, the dynamic gas treatment results showed that the respective activated carbon can successfully treat gaseous toluene under varying gas flow rates, gas concentrations and activated carbon bed depths. An increase in the carbon bed depth and decrease in toluene gas concentration and/or flow rate, yielded higher breakthrough time (BT) and exhaustion time (ET) values. Adsorption modeling employing the response surface methodology (RSM) approach successfully modeled the respective gaseous toluene removal experimental findings, with breakthrough time (BT) and exhaustion time (ET) as the response factors. The respective model-fitting parameters showed good outcomes using natural logarithmic transform model.

## 1. Introduction

An ever-growing increase in human and industrial activities geared towards a higher living standard have also caused serious environmental damage, including air pollution. To that end, gaseous emissions laden with methane [1] and toxic non-methane volatile organic compounds (NMVOC) [2,3,4] and inorganic-emissions [4,5,6,7,8] are released from many industrial and non-industrial sources. For example, gaseous NMVOC emitted from wastewater treatment plants include organic-sulfur compounds [9,10,11,12], benzene, toluene, ethylbenzene and xylene (BTEX) [10,13,14,15,16,17], fragrance compounds [18], and acetone and iso-propanol [11], whereas some inorganics include hydrogen sulfide [9], and nitrous oxide [19]. Similarly, the other major sources of NMVOC emissions include traffic, combustion, solvent evaporation, and industrial activities [2] with petroleum refineries, gas-processing plants, petrochemical plants, and specific chemical industries being the major emitters [2,20]. Unless controlled, such sites could emit toxic pollutants into the atmosphere that could potentially be transported to the nearby communities. Considering the related negative ecological and public health effects of such airborne compounds [10,15,21], several technologies have been used to reduce the related concerns [3,4,9,22]. To that end, activated carbon (AC) based adsorption also offers an efficient approach to remove several airborne pollutants. This results from AC’s high specific surface area, which gives rise to excellent adsorption efficiency, as noted for numerous gas phase pollutants including benzene [23], toluene/ethylbenzene/xylene/styrene [24], ammonia, [25,26,27,28,29], amines [30], and landfill gas laden with BTEX compounds [3]. Recently, a great interest has also been shown in AC production from otherwise waste materials [31], including agricultural by-products such as palm shells [29,32,33,34], olive stones [35], and date fruit pits [23,28], which have also been successfully used to remove gaseous pollutants. In light of the above discussion, the present work investigated the efficiency of AC derived from date palm-tree branches (DPB) agricultural waste, for gaseous toluene (C_6_H_5_CH_3_) treatment. There are several countries across the globe that practice commercial scale date fruit production [36,37] and the DPB agricultural waste from such sources can be converted to AC and used for air pollution control applications. It is important to note, that the application of DPB based AC for gaseous pollution control has not been investigated, to the best of our knowledge. Considering this, we assessed the efficiency of AC derived from DPB to treat gaseous toluene streams.

Additionally, there are several physical and chemical techniques to produce activated carbon. The surface properties of produced activated carbon including pore size are very important in determining its efficiency for the target pollutant adsorption [38,39], and such properties typically depend on the method of activated carbon production. To that end, several physical and chemical activation methods have been used [26,27,29,32] along with phosphoric acid-based activation [40]. In comparison to some chemical activation methods such as employing zinc chloride [26], the phosphoric acid activation method is environmentally friendly. Additionally, phosphoric acid-based activation has been successfully used in earlier activated carbon production studies [23,28,40]. In this work, we evaluated the effect of several operational variables, including gas flow rate, and gas concentration on the ability of phosphoric acid activated DPB based AC to remove gaseous toluene under dynamic flow conditions. The experimental results were modeled using the response surface methodology (RSM) approach, with details given in the coming sections.

## 2. Materials and Methods

### 2.1. Materials

The materials used were 200 ppmv toluene standard gas, pH 4, 7, and 10 standard-solutions (FISHER), monopotassium phosphate (*w*/*w* 85%; BAKER), and zero air (Zero Air Setup, Thermo Scientific, Waltham, MA, USA).

### 2.2. Preparation Procedure for Activated Carbon

Local date palm-tree branches (DPB) were first crushed (CB), cleaned thoroughly, and then kept for a day at 120 °C in an oven. After this step, the respective crushed DPB material was mixed with 40% *w/w* phosphoric acid at an impregnation ratio/R of 2 (R = (mL-acid/gm-CB)) and then placed in an oven for overnight. The resulting slurry was kept in stainless steel tubes (length 30 cm; dia 2.5 cm) with exhaust holes, and those tubes were transferred to a furnace (Lenton, Hope Valley, UK) and its temperature was gradually adjusted to 700 °C followed by 1 h retention time. The obtained mass was washed till pH ≈ 7.0 followed by drying at 120 °C and storage till further use.

### 2.3. Toluene Gas Treatment

**Figure 1:**
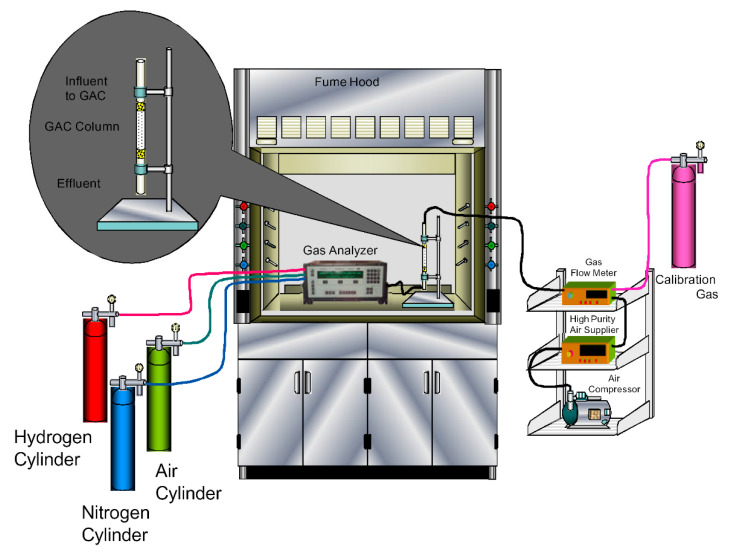
The experimental setup used for the dynamic continuous toluene gas flow adsorption experiments.

All toluene gas treatment tests were performed at room temperature and atmospheric pressure, employing a setup as outlined in Figure 1. The respective dynamic adsorption studies were conducted using 200 ppmv toluene calibration standard that was diluted with high-purity zero air. During each adsorption study, the pollutant gas (at a specific concentration and flow rate) was continuously passed through a column setup (FEP tubing with 6.35 mm inner diameter) holding the respective DPB derived activated carbon (Figure 1). The influent/effluent toluene gas concentration was quantified using the state-of-the-art gas analyzer (Thermo Scientific, Waltham, MA, USA).

### 2.4. Analytical Methods

The produced DPB derived AC was characterized by N_2_ adsorption/desorption isotherm for determining the BET specific surface area (*SSA*_BET_), pore volume, pore area and pore width (Micromeritics, Norcross, GA, USA). The FTIR data for produced AC were obtained between 4000 to 600 cm^−1^ for determining the surface functional groups (Thermo Scientific, Waltham, MA, USA). Furthermore, the respective thermogravimetric analysis was completed up to temperature of 1000 °C under N_2_ gas flow to realize the thermal stability of the DPB AC (Perkin Elmer TGA 4000 analyzer). The TGA measurements were conducted by increasing the temperature in the chamber to 105 °C, holding it for 3 min and then increasing up to 1000 °C at 15°C/min. The gaseous toluene was analyzed using non-methane hydrocarbon analyzer (Thermo Scientific, Waltham, MA, USA).

### 2.5. Response Surface Methodology (RSM) Modeling

For the response surface methodology (RSM) modeling, the influent gas flow rate, influent gas concentration, and activated carbon column/bed depth, were selected as the factors and their respective levels are shown in Table 1. Furthermore, the RSM design used in the present was based on the face-centered central composite design (FC-CCD) type with one center point and the respective experimental design is given in Table 2. The breakthrough (BT) and exhaustion times (ET) were selected as the responses, for which the model equations were developed. The breakthrough time denotes the time (during a dynamic continuous gas flow adsorption study) at which the effluent gas concentration reaches 5% of the influent gas concentration, whereas the exhaustion time denotes the time at which the effluent gas concentration reaches 95% of the influent gas concentration. The gas flow rates are given in standard liters per minute (slpm).

## 3. Results and Discussion

### 3.1. Activated Carbon (AC) Characterization

**Figure 2:**
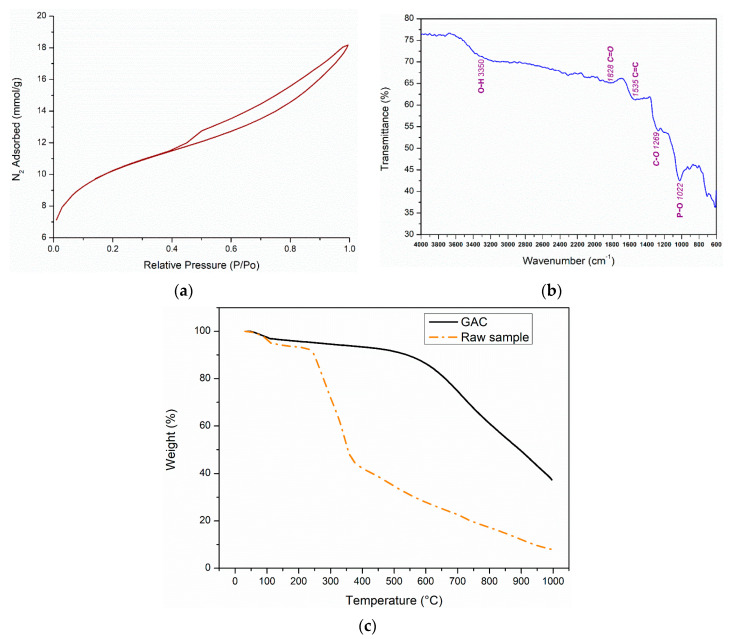
(**a**) The BET adsorption isotherm findings for the date palm-tree branches activated carbon (AC) (preparation conditions: 40% *w/w* phosphoric acid; impregnation ratio/R of 2 (R = (mL-acid/gm-CB)); Furnace temperature 700 °C for 1 h). (**b**) The transmittance vs. wavenumber Fourier transform infrared spectroscopy (FTIR) findings for the produced AC (preparation conditions: 40% *w/w* phosphoric acid; impregnation ratio/R of 2 (R = (mL-acid/gm-CB)); furnace temperature 700 °C for 1 h). (**c**) TGA curves for the produced AC and raw CB (preparation conditions for AC: 40% *w/w* phosphoric acid; impregnation ratio/R of 2 (R = (mL-acid/gm-CB)); Furnace temperature 700 °C for 1 h). CB: crushed date palm-tree branches.

Several characterization results for the DPB based AC are provided in Figure 2a–c. Figure 2a shows the BET isotherm for the DPB AC. According to IUPAC classification, the BET isotherms are classified into six types based on the shape of the BET isotherm and accordingly the material pore distribution is classified as (a) <2 nm (micropores), (b) between 2 and 50 nm (mesopores), and (c) >50 nm (macropores) [23,38]. The BET curve for DPB AC in Figure 2a displays a hysteresis loop from 0.4 P/P_o_ which is due to capillary condensation in the mesoporous structure [38,39]. The curve can be classified as a type IV isotherm which is characteristic of materials having a micro- and mesoporous structure [39]. The BET specific surface area was found to be 800.87 m^2^/g, whereas the average pore width of 30.32 Å is in the mesoporous range (Table 3). These findings suggest that the DPB AC is highly porous consisting of both micro and mesopores. Additionally, the production of respective activated carbon at 500 °C, R = 2, and using 20% phosphoric acid, yielded a specific surface area of 646 m^2^/g. Additionally, under same conditions, and increasing temperature to 600 °C, yielded an activated carbon specific surface area of 748 m^2^/g. On the other hand, at an impregnation ratio of 2.4, 20% phosphoric acid, and temperatures 600 and 700 °C, the specific surface area values were 701 and 768 m^2^/g, respectively. Hence, the activated carbon produced at an impregnation ratio of 2, 40% phosphoric acid, and 700 °C, shows a higher specific surface area.

Figure 2b shows the FTIR findings for the produced DPB AC. The bands between 900 cm^−1^ to 1400 cm^−1^ are generally designated to C-N, C-O, S = O and C-H surface groups and the peak at 1022 cm^−1^ represents bending vibrations due to P-O bond stretching [23]. The bands between 1450–1550 cm^−1^ are ascribed to C = C stretching and are indicative of the presence of aromatic rings, arising due to an increase in the aromaticity during the activation process, and the broad band at around 3350 cm^−1^ is designated to O-H groups [40]. The peaks between 1700–1900 cm^−1^ are designated to C = O vibrations of carboxylic groups, aldehydes and ketones [41]. These surface functional groups (SFG) can significantly affect the adsorption of toluene onto DPB AC considering the possible interactions of the ring structure of toluene with the surface functional groups containing oxygen [23].

The TGA profiles of the DPB AC and raw date palm branches are displayed in Figure 2c. The weight loss trends, as observed for the activated carbon and raw crushed date palm-tree branch (CB) samples are similar to those reported by Vohra [23] for AC produced from date pits. After the initial moisture loss observed at temperatures up to 105 °C, the TGA profile of the raw CB sample displays a gradual weight loss until 250 °C after which there is a sharp decrease in the weight until 350 °C, followed again by a gradual change. The gradual decrease until 250 °C is indicative of hydrocarbon removal, whereas the sharp decrease after 250 °C results from the loss of lignin- and cellulose-based materials. Additionally, the noted decrease after 350 °C results from the oxidation of residual carbon. In comparison, the TGA profile of DPB AC shows that after the initial moisture loss, the DPB AC is stable until a temperature of about 550 °C after which there is a sharp loss in weight continuing to 1000 °C. The insignificant loss of weight until 550 °C is mainly due the activation process, which led to the degradation of lignin- and cellulose-based components during the DPB AC synthesis. The loss in weight after 550 °C results from the oxidation of DPB AC [23]. The total weight loss is 62.7 and 92% for DPB AC and raw CB, respectively. In summary, the characterization results reveal that DPB AC consist of both micro- and mesopores, with a high *SSA*_BET_ with several surface functional groups.

### 3.2. Effect of Operational Parameters on Toluene Gas Adsorption

**Figure 3:**
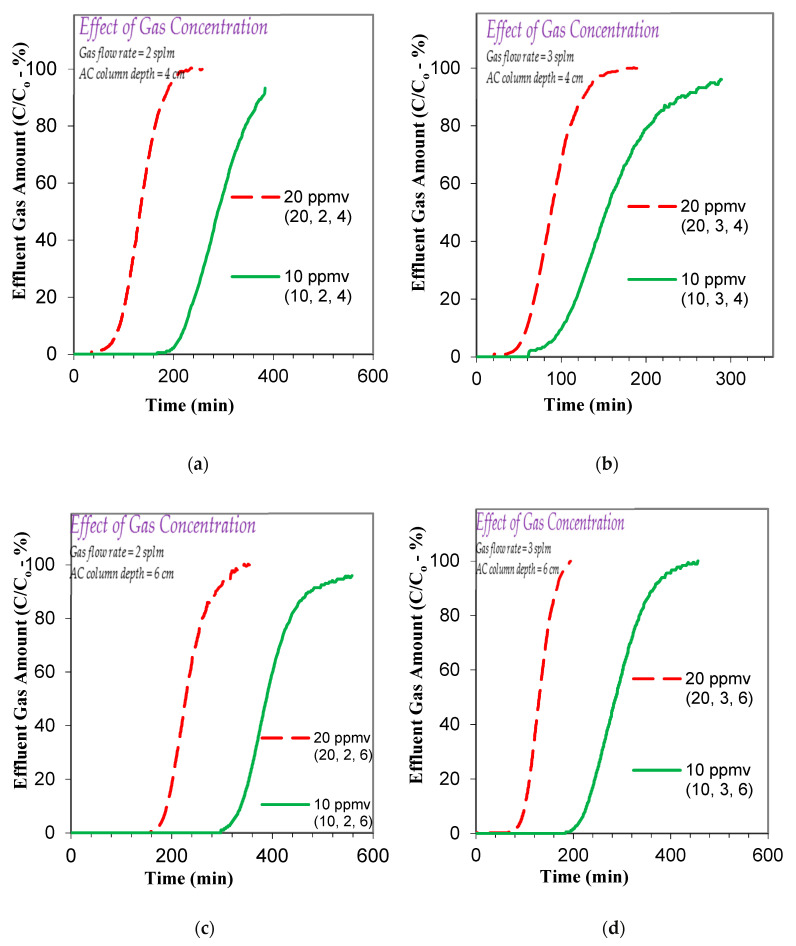
Effect of influent toluene/C_6_H_5_CH_3_ gas concentration on the adsorption breakthrough curve profile of toluene/C_6_H_5_CH_3_ gas using AC produced from date palm-tree branches (**a**) toluene/C_6_H_5_CH_3_ gas flow rate flow rate = 2 slpm; influent gas concentrations 10 and 20 ppmv; AC bed depth = 4 cm; (**b**) toluene/C_6_H_5_CH_3_ gas flow rate flow rate = 3 slpm; influent gas concentrations 10 and 20 ppmv; AC bed depth = 4 cm; (**c**) toluene/C_6_H_5_CH_3_ gas flow rate flow rates = 2 slpm; influent gas concentrations 10 and 20 ppmv; AC bed depth = 6 cm; (**d**) toluene/C_6_H_5_CH_3_ gas flow rate flow rate = 3 slpm; influent gas concentrations 10 and 20 ppmv; AC bed depth = 6 cm; (AC *SSA*_BET_ = 800.87 m^2^/g and average pore width = 30.32 Å; AC bed dia. 6.35 mm).

The present study first investigated the effect of toluene gas concentration on its adsorption-based removal, and to that end, a series of experiments were conducted at different influent toluene gas concentrations (20 ppmv and 10 ppmv) using column lengths of 4 and 6 cm and gas flow rates of 2 and 3 slpm. The overall results, as depicted in Figure 3a–d, show that the breakthrough and the exhaustion time values increase with a decrease in the influent toluene gas concentration. At an AC column/bed depth of 4 cm and flow rate of 2 slpm, a decrease in the influent toluene gas from 20 to 10 ppmv increases the breakthrough from 80 to 211 min and the exhaustion from 195 min to 384 min (Figure 3a). This is also the case for a higher flow rate of 3 slpm wherein the breakthrough goes up from 53 to 86 min and the exhaustion from 137 to 286 min, as the concentration decreases from 20 to 10 ppmv (Figure 3b). Furthermore, qualitatively similar (to the above mentioned 4 cm AC column) trends are observed for the 6 cm AC bed depth while keeping the other parameters the same, as shown in Figure 3c,d. It is noted that the breakthrough time values increase from 183 to 324 min at 2 slpm and from 93 to 215 min at 3 slpm, whereas the exhaustion time values increase from 307 to 543 min at 2 slpm and from 175 to 390 min at 3 slpm, as shown in Figure 3c,d. Furthermore, the respective increases in breakthrough and exhaustion times are approximately proportional to the respective decrease in the influent toluene gas concentration, which suggests that the pore and bulk diffusion processes are sufficiently faster, yielding significant overall mass transfer to the AC, even at 20 ppmv toluene gaseous concentration.

**Figure 4:**
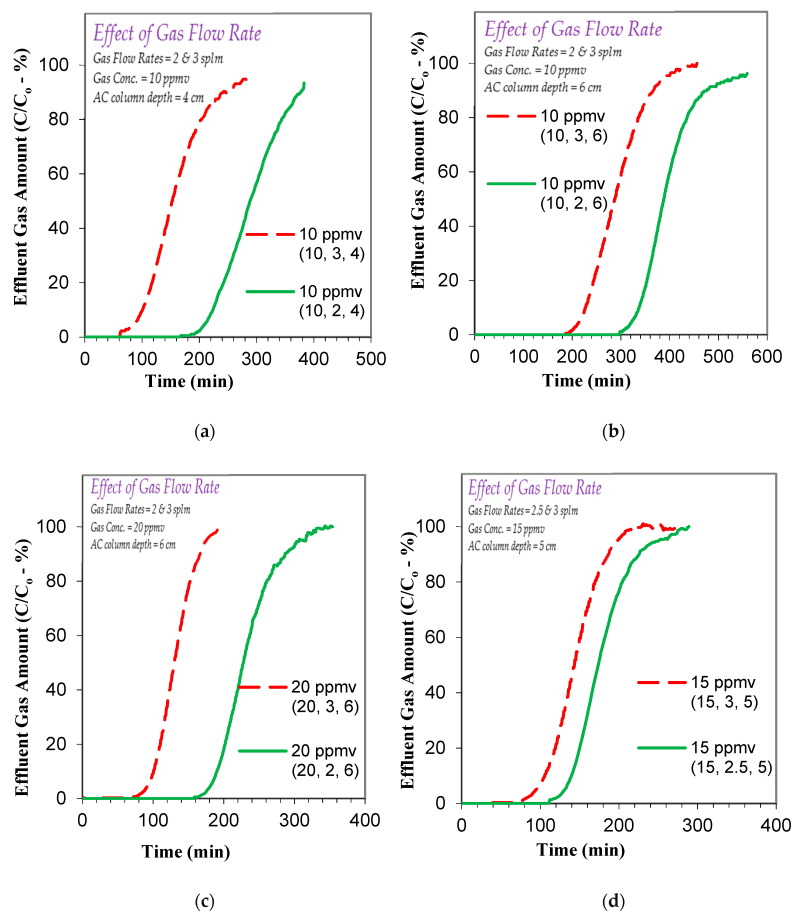
Effect of influent toluene/C_6_H_5_CH_3_ gas flow rate on the adsorption breakthrough curve profile of toluene/C_6_H_5_CH_3_ gas using AC produced from date palm-tree branches (**a**) toluene/C_6_H_5_CH_3_ gas flow rate flow rates = 2 and 3 slpm; influent gas concentration 10 ppmv; AC bed depth = 4 cm; (**b**) toluene/C_6_H_5_CH_3_ gas flow rate flow rates = 2 and 3 slpm; influent gas concentration 10 ppmv; AC bed depth = 6 cm; (**c**) toluene/C_6_H_5_CH_3_ gas flow rate flow rates = 2 and 3 slpm; influent gas concentration 20 ppmv; AC bed depth = 6 cm; (**d**) toluene/C_6_H_5_CH_3_ gas flow rate flow rates = 2.5 and 3 slpm; influent gas concentration 15 ppmv; AC bed depth = 5 cm; (AC *SSA*_BET_ = 800.87 m^2^/g and average pore width = 30.32 Å; AC bed dia. 6.35 mm).

To build on the above given findings, the effect of gas flow rate on to removal of toluene was also studied and respective results are summarized in Figure 4a–d. For an influent toluene concentration of 10 ppmv and 4 cm AC column length, an increased flow from 2 to 3 slpm leads to a decrease in the breakthrough and exhaustion times from 211 to 86 min and from 384 to 286 min, respectively (Figure 4a). Additionally, for a higher column length of 6 cm and influent toluene concentration of 10 ppmv (Figure 4b), the lowering in the breakthrough and exhaustion values upon increasing the flow rate from 2 to 3 slpm is noted to be from 324 min to 215 min and from 543 min to 390 min, respectively. Similarly, for 20 ppmv toluene and a 6 cm AC column, an increase in gas flow from 2 to 3 slpm causes the breakthrough to decrease from 183 to 93 min, while the exhaustion time decreases from 307 to 175 min, as shown in Figure 4c. Furthermore, at a toluene concentration of 15 ppmv (for 5 cm AC column depth), an increase in gas flow from 2.5 to 3 slpm causes the breakthrough to decrease from 131 to 95 min while the exhaustion time decreases from 262 to 199 min (Figure 4d). The lowering of respective breakthrough and exhaustion values with an increase in the influent toluene gaseous flow rate is indicative of fixed surface adsorption sites. Hence, for the complexation of toluene at the AC surface, an increase in the influent gas results in the available adsorption sites being consumed faster, which causes a decrease in both the breakthrough and exhaustion times.

**Figure 5:**
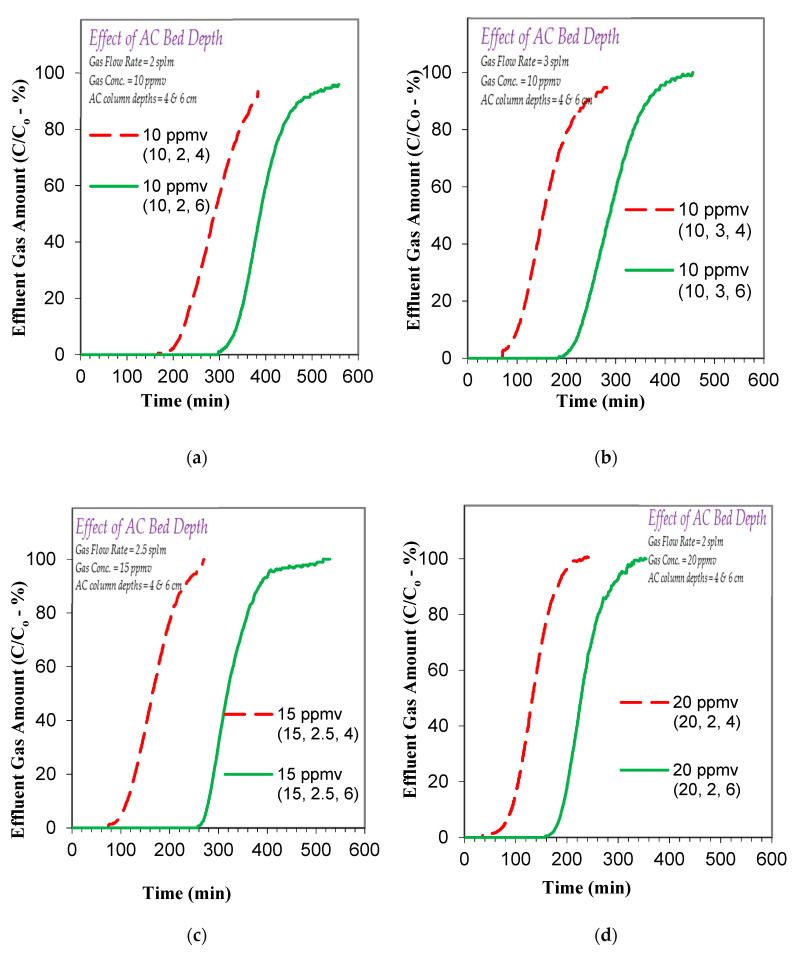
Effect of AC column length on the adsorption breakthrough curve profile of toluene/C_6_H_5_CH_3_ gas on to AC produced from date palm branches (**a**) toluene/C_6_H_5_CH_3_ gas flow rate flow rate = 2 slpm; influent gas concentration 10 ppmv; AC column lengths = 4 and 6 cm; (**b**) toluene/C_6_H_5_CH_3_ gas flow rate flow rates = 3 slpm; influent gas concentrations 10 ppmv; AC column lengths = 4 and 6 cm; (**c**) toluene/C_6_H_5_CH_3_ gas flow rate flow rate = 2.5 slpm; influent gas concentration 15 ppmv; AC column lengths = 4 and 6 cm; (**d**) toluene/C_6_H_5_CH_3_ gas flow rate flow rate = 2 slpm; influent gas concentration = 20 ppmv; AC column lengths = 4 and 6 cm; (AC *SSA*_BET_ = 800.87 m^2^/g and average pore width = 30.32 Å; AC bed dia. 6.35 mm).

After studying the effects of toluene gas concentration and flow rate, the AC bed depth (cm) effect on to toluene removal was also assessed at toluene influent concentrations of 10, 15 and 20 ppmv and toluene flow rates of 2, 2.5 and 3 slpm for column lengths of 4 and 6 cm. The respective results, as given in Figure 5a–d, show that an increase in the column length from 4 to 6 cm leads to a simultaneous increase in both breakthrough and exhaustion times. For example, the breakthrough time is noted to increase from 211 to 324 min for the 10-ppmv/2-slpm system (Figure 5a), from 86 to 215 min for the 10-ppmv/3-slpm system (Figure 5b), from 102 to 274 min for the 15-ppmv/2.5-slpm system (Figure 5c), and from 80 to 183 min for the 20-ppmv/2-slpm system (Figure 5d). Similarly, the exhaustion time increases (due to respective increase in column length) from 384 to 543 min for the 10-ppmv/2-slpm system (Figure 5a), from 286 to 390 min for 10-ppmv/3-slpm system (Figure 5b), from 249 to 405 min for 15-ppmv/2.5-slpm system (Figure 5c), and 195 to 307 min for 20-ppmv/2-slpm system (Figure 5d). Thus, longer column lengths enhance both the breakthrough and exhaustion times. These findings are similar to earlier studies on the removal of gaseous ammonia and benzene using activated carbon produced from date fruit pits [23,28]. The increase in the breakthrough and exhaustion times with an increased AC column length is mainly due to the availability of more adsorbent based surface complexation sites, which in turn results in higher adsorption of gaseous toluene. A brief summary of the results is presented in Table 4. It is also typically noted that the effect of increasing the column length is qualitatively more pronounced at higher toluene gas concentrations. Additionally, the toluene concentration effect results (Figure 3) indicated that the diffusion processes are sufficiently faster, yielding significant overall mass transfer to the AC even at a 20 ppmv toluene gaseous concentration. A similar effect has been reported earlier for toluene adsorption onto AC, i.e., the diffusion coefficient increases with an increase in the initial toluene concentration [42]. On the other hand, the surface complexation of toluene should also play an important role in its overall adsorption on the AC surface. It is possible that both hydrogen bonding and the van der Waals interactions, between the toluene gas molecules and the AC surface can initiate adsorption of toluene [23,34,43,44]. These interactions between the toluene molecules and AC surface are proposed to be initiated by potential DPB AC surface functional groups, as discussed in Section 3.1, including S=O, C-O, C=O, P-O etc. The above-mentioned interactions can be summarized as:C_6_H_5_CH_3_ (bulk-gas) 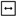 C_6_H_5_CH_3_ (interface)(1)
C_6_H_5_CH_3_ (interface) 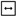 C_6_H_5_CH_3_ (bulk-solid)(2)
GAC-O^−^ + C_6_H_5_CH_3_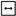 GAC-O-C_6_H_5_CH_3_(3)

It is proposed that the DPB AC surface functional groups do provide the potential sites for toluene adsorption. Furthermore, in the case of the present study, the stated pore volume for DPB AC, i.e., 0.437 cm^3^/g (Table 3), is similar to other AC based toluene gas removal studies where both AC surface-chemistry and porosity were noted to be important for toluene adsorption capacity [11,43,45,46,47,48].

### 3.3. RSM Modeling

Table 1 shows the factors and their levels and Table 2 shows the (FC-CCD) experimental design considered for developing the RSM model. The models were developed considering two responses i.e., breakthrough time and exhaustion time. The breakthrough time denotes the time at which the toluene effluent concentration was 5% of the influent gas concentration whereas the exhaustion time denotes the time at which the toluene effluent concentration was 95% of the influent gas concentration. The gas flow rates are given in standard liters per minute (slpm).

#### 3.3.1. Toluene Gas Adsorption Breakthrough Time and Exhaustion Time Models

Table 5 and Table 6 show the Significance related RSM modeling details for the toluene breakthrough and exhaustion, respectively. The breakthrough time (BT) and the exhaustion time (ET) models were transformed using a natural logarithmic function, which significantly improved the model parameters as compared to the untransformed data. The models are represented by Equations (4) and (5) for the toluene breakthrough and exhaustion time, respectively. The respective model-fitting statistics are also provided in Table 7.

The model *p*-values for the toluene breakthrough and exhaustion are provided in Table 5; Table 6, respectively. Generally, *p*-values < 0.05 (for factors used in modeling) indicate that the respective factors are significantly contributing towards improving the modeling results whereas the terms having *p*-values more than 0.1 are generally considered insignificant [49]. For the present study, all the terms (i.e., A, B and C) have *p*-values of less than 0.05 (Table 5 and Table 6) and are thus considered significant for the respective RSM models.

*ln(BT)* = 6.07811 − 0.687414*A* − 0.372310*B* − 0.086222*C* (4)

where *BT* = toluene gas breakthrough time @ 5% (min); *A* = toluene gas flow rate (slpm); *B* = activated carbon column depth (cm); *C* = toluene gas concentration (ppmv).

*ln(ET)* = 6.98428 − 0.465006*A* − 0.184171*B* − 0.072338*C* (5)

where *ET* = toluene gas exhaustion time @ 95% (min).

**Figure 6:**
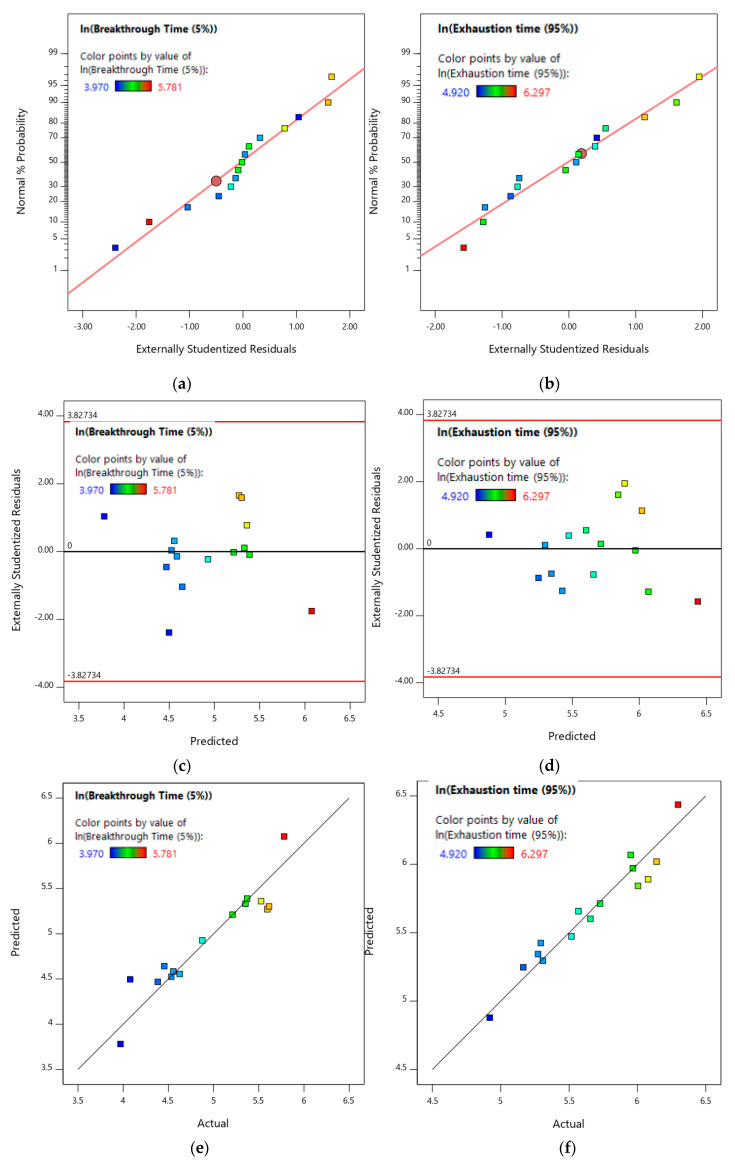
(**a**) Normal plot of residuals for reduced quadratic toluene breakthrough model. (**b**) Normal plot of residuals for reduced quadratic toluene exhaustion model. (**c**) Residual vs. predicted plot for reduced quadratic toluene breakthrough model. (**d**) Residual vs. predicted plot for reduced quadratic toluene exhaustion model. (**e**) Predicted vs. actual plot for reduced quadratic toluene breakthrough model. (**f**) Predicted vs. actual plot for reduced quadratic toluene exhaustion model.

The predicted and adjusted R^2^ values for the breakthrough model are 0.7982 and 0.8524, respectively, and the respective exhaustion model parameters are 0.8845 and 0.9168, respectively. Both sets of predicted and adjusted R^2^ values with a difference of less than 0.2, show a reasonable model fit. Furthermore, the adequate precision values of 19.3374 and 25.5566 for the breakthrough and exhaustion parameters, respectively, which are >4, are also indicative of a good model fit. The respective models were also analyzed based on the assumptions of normality and random variation of the residuals (i.e., any deviation from these assumptions will invalidate the models). Figure 6a,b shows the normal plot of residuals for the toluene breakthrough and exhaustion, respectively, and Figure 6c,d shows the residual vs. predicted plot for toluene breakthrough and exhaustion models, respectively. As seen in Figure 6a,b, the residuals closely follow the normal distribution line and as such the assumption of normality of residuals is satisfied. Additionally, from Figure 6c,d, it can be observed that the residuals are randomly distributed and no pattern can be observed satisfying the assumption of randomness of residuals. Hence, the respective normality and randomness results indicate that the toluene breakthrough and exhaustion models are valid.

Figure 6e,f shows the model predictions vs. actual experimental values for toluene breakthrough and exhaustion, respectively. The 45° line-slope in both Figures shows that the model predictions closely match with the respective experimental results. The Predicted R^2^ value of 0.7982 and 0.8845 suggests that the model predictions are in good agreement with the toluene exhaustion model, showing better prediction accuracy. The higher Predicted R^2^ value for the toluene exhaustion time model might be due to fact that the exhaustion times are higher than the breakthrough times and hence have less variability as initial adjustments might lead to higher variability in the breakthrough model. In general, the RSM model analysis closely matched with the experimental findings, as shown in Figure 6e,f.

#### 3.3.2. Effect of Factors on Toluene Gas Adsorption Breakthrough and Exhaustion Times

**Figure 7:**
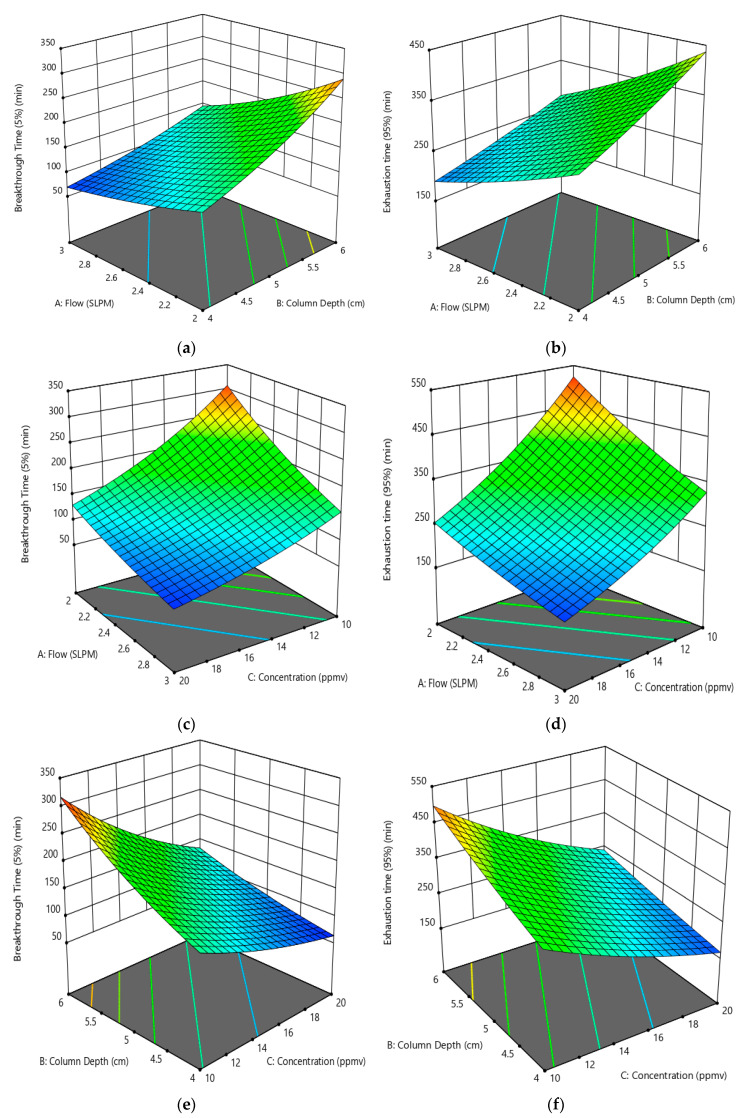
Response surface profiles showing the variation of (**a**) toluene breakthrough times (min) with toluene flow rate (slpm) and column depth (cm) (influent toluene concentration 15 ppmv). (**b**) Toluene exhaustion times (min) with toluene flow rate (slpm) and column depth (cm) (influent toluene concentration 15 ppmv). (**c**) Toluene breakthrough times (min) with toluene flow Rate (slpm) and influent toluene concentration (ppmv) (column depth 5 cm). (**d**) Toluene exhaustion times (min) with toluene flow rate (slpm) and influent toluene concentration (ppmv) (column depth 5 cm). (**e**) Toluene breakthrough times (min) with column depth and influent toluene concentration (ppmv) (toluene flow rate 2.5 slpm). (**f**) Toluene exhaustion times (min) with column depth and influent toluene concentration (ppmv) (toluene flow rate 2.5 slpm).

Figure 7a–e shows the variation of toluene breakthrough time (BT) and exhaustion time (ET) with the factors studied, i.e., toluene gas flow rate, depth of granular activated carbon column and influent toluene gas concentration. Figure 7a,b shows the effect of gas flow rate and column depth on toluene breakthrough and exhaustion, respectively, at 15 ppmv influent toluene. The respective breakthrough and exhaustion times decrease with an increase in the flow rate from 2 to 3 slpm. The reduction in breakthrough and exhaustion values at higher flow rates results because of higher toluene molecules adsorption per unit time on to GAC surface, thus, leading to reduced breakthrough and exhaustion times. Although the breakthrough and exhaustion times are less for the lower column depths, the variations with flow rate are qualitatively similar at the two depths. For example, the BT and ET increase from 69 to 137 min and from 190 to 302 min for the 4 cm column depth, respectively. On the other hand, the BT and ET increase from 145 to 290 min and from 275 to 437 min for the 6 cm column depth, respectively.

Figure 7c,d shows the variation of breakthrough and exhaustion time with the influent toluene concentration and toluene flow rate at a fixed column depth of 5 cm. It can be observed from Figure 7c, that as the influent toluene concentration is increased from 10 to 20 ppmv, the breakthrough time decreases from 307 to 130 min and from 155 to 65 min for flow rates 2 and 3 slpm, respectively. Similarly, the results in Figure 7d also show that as the influent toluene concentration is increased from 10 to 20 ppmv, the exhaustion time reduces from 522 to 255 min and from 328 to 159 min for flow rates 2 and 3 slpm, respectively. The respective reductions are indicative of fixed adsorption sites and specific surface area of the GAC and thus, an increase in the influent gas concentration decreases both the breakthrough and exhaustion times.

Figure 7e,f shows the variation in toluene breakthrough and exhaustion times with the column depth and influent toluene gas concentration for a flow rate of 2.5 slpm. These figures reveal that upon increasing the activated carbon column depth from 4 cm to 6 cm, the toluene breakthrough time increased from 137 to 290 min and 70 to 146 min, whereas the exhaustion time increased 300 to 437 min and 190 to 275 min for toluene gas influent concentrations of 10 and 20 ppmv, respectively. These findings suggest that although the increase in column depth leads to an increase in the empty bed contact time (EBCT), there is also an increase in the throughput, i.e., the bed volumes of toluene gas treated before the breakthrough occurs, which results in higher breakthrough and exhaustion times. The above findings indicate successful abatement of gaseous toluene by employing AC derived from DPB, under different operating conditions.

## 4. Conclusions

Treating toxic industrial gaseous pollutants is important both to maintain public health and to minimize negative impacts on the environment and ecology. To that end, the present findings indicate that activated carbon (AC) produced from date palm-tree branches (DPB) can successfully treat gaseous toluene (C_6_H_5_CH_3_) emissions. We evaluated the effect of several operational variables including gas flow rate, gas concentration and AC bed depth, on the efficiency of respective AC to remove gaseous toluene under dynamic flow conditions. The respective findings indicate that the DPB based AC can effectively treat gaseous toluene (C_6_H_5_CH_3_), under different operating conditions. It was noted that a decrease in the gas flow rate and concentration, and an increase in the AC bed depth, led to an increase in both the breakthrough and exhaustion times. This was investigated at various operating conditions and the results indicated that the breakthrough and exhaustion time values showed similar trends at different operating conditions. Furthermore, the response surface methodology (RSM) approach was also used to model toluene (C_6_H_5_CH_3_) gas removal with breakthrough time (BT) and exhaustion time (ET) as the response factors. The experimental results fitted well with the models developed using the RSM approach. The model for breakthrough time had an adjusted R^2^ value of 0.8524 and a predicted R^2^ value of 0.7982 with an adequate precision of 19.3374, whereas the model for exhaustion time had an adjusted R^2^ value of 0.9168 and a predicted R^2^ value of 0.8845 with an adequate precision of 25.5566. These parameters indicate that the respective RSM models have a good prediction accuracy. It is also noted that the exhaustion time model has higher adjusted and predicted R^2^ values as compared to the breakthrough time model. This might be because the exhaustion times are higher than the breakthrough times and hence, have less variability as initial adjustments might lead to higher variability in the breakthrough model. In general, the present findings indicate that AC from DPB agricultural waste can be successfully used for the treatment of gaseous toluene under a varying set of operational conditions. Based on the above-mentioned promising outcomes, this work will be expanded in the future to investigate the other BTEX gaseous pollutants, in addition to a comparison with a commercial activated carbon and the re-generated DPB based activated carbon.

## Figures and Tables

**Table 1:** Factors and their levels for toluene adsorption response surface methodology (RSM) modeling.

Factors	Level −1	Level 0	Level 1
A = Toluene Gas Flow Rate (slpm)	2	2.5	3
B = AC Bed Depth (cm)	4	5	6
C = Toluene Gas Concentration (ppmv)	10	15	20

**Table 2:** Design Table for toluene Adsorption RSM Modeling.

Exp No.	Factor A: Influent Toluene Gas Flow Rate (slpm)	Factor B: GAC Bed Depth (cm)	Factor C: Influent Toluene Gas Concentration (ppmv)
1	2	5	15
2	3	6	20
3	2	4	10
4	2.5	4	15
5	2.5	6	15
6	2	4	20
7	3	4	10
8	2	6	10
9	2.5	5	20
10	2	6	20
11	3	4	20
12	2.5	5	10
13	2.5	5	15
14	3	6	10
15	3	5	15

**Table 3:** *SSA*_BET_ properties of date palm-tree branches (DPB) based activated carbon (AC).

Property	Value
*SSA*_BET_	800.87 m^2^/g
t-Plot Micropore Area	335.25 m^2^/g
t-Plot External Surface Area	465.62 m^2^/g
t-Plot Micropore Volume	0.150 cm^3^/g
Total Pore Volume	0.437 cm^3^/g
Average Pore Width (4 V/A by BET)	30.32 Å

**Table 4:** Summarized effects of operating parameters on to toluene adsorption efficiency.

Parameter Changed	Change in Parameter	Change in Breakthrough Time Observed	Change in Exhaustion Time Observed	Reason
Influent Gas Concentration	Increased 10 to 20 ppmv	Decreased	Decreased	Fixed adsorption sites on the GAC surface
Flow Rate	Increased 2 to 3 slpm	Decreased	Decreased	Faster consumption of adsorption sites at higher flow rates
Column Depth	Increased 4 to 6 cm	Increased	Increased	Availability of more adsorbent based surface complexation sites

**Table 5:** Significance related details for the logarithmic linear toluene breakthrough RSM model.

Source	*p*-Value	Significance
Model	<0.0001	Significant
A: Toluene Gas Flow Rate (slpm)	0.0006	Significant
B: Activated Carbon Column Depth (cm)	0.0003	Significant
C: Toluene Gas Concentration (ppmv)	<0.0001	Significant

**Table 6:** Significance related details for the logarithmic linear toluene exhaustion RSM model.

Source	*p*-Value	Significance
Model	<0.0001	significant
A: Toluene Gas Flow Rate (slpm)	<0.0001	significant
B: Activated Carbon Column Depth (cm)	0.0004	significant
C: Toluene Gas Concentration (ppmv)	<0.0001	significant

**Table 7:** Model-fitting statistical details for logarithmic linear toluene breakthrough and exhaustion models.

Statistical Parameter	Response	
	Breakthrough Time	Exhaustion Time
R^2^	0.8840	0.9346
Adjusted R^2^	0.8524	0.9168
Predicted R^2^	0.7982	0.8845
Adequate Precision	19.3374	25.5566
